# Role of Non-coding RNAs in Axon Regeneration after Peripheral Nerve Injury

**DOI:** 10.7150/ijbs.70290

**Published:** 2022-05-09

**Authors:** Ming Liu, Pei Li, Yuanyuan Jia, Qingjun Cui, Kexin Zhang, Jingjing Jiang

**Affiliations:** Department of Anesthesiology, Shengjing Hospital of China Medical University, Shenyang, China

**Keywords:** non-coding RNA, axon regeneration, peripheral nervous system, Schwann cells, peripheral nerve injury

## Abstract

Peripheral nerve injury (PNI) may lead to disability and neuropathic pain, which constitutes a substantial economic burden to patients and society. It was found that the peripheral nervous system (PNS) has the ability to regenerate after injury due to a permissive microenvironment mainly provided by Schwann cells (SCs) and the intrinsic growth capacity of neurons; however, the results of injury repair are not always satisfactory. Effective, long-distance axon regeneration after PNI is achieved by precise regulation of gene expression. Numerous studies have shown that in the process of peripheral nerve damage and repair, differential expression of non-coding RNAs (ncRNAs) significantly affects axon regeneration, especially expression of microRNAs (miRNAs), long non-coding RNAs (lncRNAs) and circular RNAs (circRNAs). In the present article, we review the cellular and molecular mechanisms of axon regeneration after PNI, and analyze the roles of these ncRNAs in nerve repair. In addition, we discuss the characteristics and functions of these ncRNAs. Finally, we provide a thorough perspective on the functional mechanisms of ncRNAs in nervous injury repair, and explore the potential these ncRNAs offer as targets of nerve injury treatment.

## Introduction

Injuries of the central and peripheral nervous system are common in clinical practice. Severe nerve injury has a destructive impact on patients' quality of life. Typical symptoms are sensory and motor function deficits, which can result in complete paralysis of the affected limb or development of intractable neuropathic pain 1. Neuropathic pain is caused by pathological changes or injury of the peripheral nerve or central sensory conduction pathway. After nerve injury, neuropathic pain is closely related to nerve regeneration and repair 2. Due to lack of an inherent ability to regenerate, as well as the inhibitory effect of the extracellular barrier, neurons in the central nervous system (CNS) are incapable of regeneration 3. In contrast, neurons in the peripheral nervous system (PNS) show strong regenerative capacity 4. In the PNS, functional recovery can often occur following injury since neurons have the capacity for self-repair and can reactivate intrinsic growth programs 5.

Currently, the treatment of choice for peripheral nerve injury (PNI) is advanced microsurgical end-to-end repair with tensionless epineurial sutures, autologous nerve grafting and nerve transfer where end-to-end anastomosis is not possible 6,7. Less than half of patients who undergo nerve repair after injury regain good to excellent motor or sensory function 8, and up to one-third of patients may have little or no recovery despite appropriate surgical intervention 9. Recently, growth factor (GF) supplementation has been developed as a new multifunctional treatment strategy which can promote nerve regeneration and functional recovery. However, due to the short half-life of GFs and their rapid inactivation in body fluids, administration of GFs alone is not sufficient for repairing PNI 10. Therefore, researchers urgently need to develop new therapeutic or adjunctive strategies to promote functional recovery in PNI patients. With the progress made by researchers in nerve regeneration after PNI, epigenetic regulation at the gene level has attracted greater attention.

The regulatory effect of non-coding RNAs (ncRNAs) on the expression of genes related to regeneration after PNI may provide a new target for axon regeneration after nerve injury. In this review, we summarize the cellular and molecular mechanisms of axon regeneration after PNI, introduce ncRNAs and focus on some ncRNAs that are critical for neuron regeneration. We also offer suggestions for future studies and treatment strategies in this field.

## Molecular mechanisms of axon regeneration

After nervous system injury, the main internal factors that determine the inherent growth ability of axons are the expression and mobilization of axon regeneration-associated genes in nerve cells and the availability of cytoskeletal raw materials. The realization of axon regeneration requires the coordination of a variety of biological processes, including but not limited to recognition of cell damage, gene transcription, biological macromolecule synthesis, axoplasmic transport, cell energy homeostasis maintenance and cytoskeleton assembly. The transition to active neuronal regeneration is usually driven by the transcriptome, in which transcription factors (TFs) are responsible for coordinating the expression of multiple regeneration-associated genes 11. These TFs include ATF3, Smad1 and STAT3 among others 12-14. Regulation of these factors can effectively change neuronal transcription and promote regeneration. Epigenetic modification regulates gene transcription and can affect nerve regeneration by controlling ncRNAs, histone modification or DNA modification without altering the DNA sequence 15.

**Figure 1:**
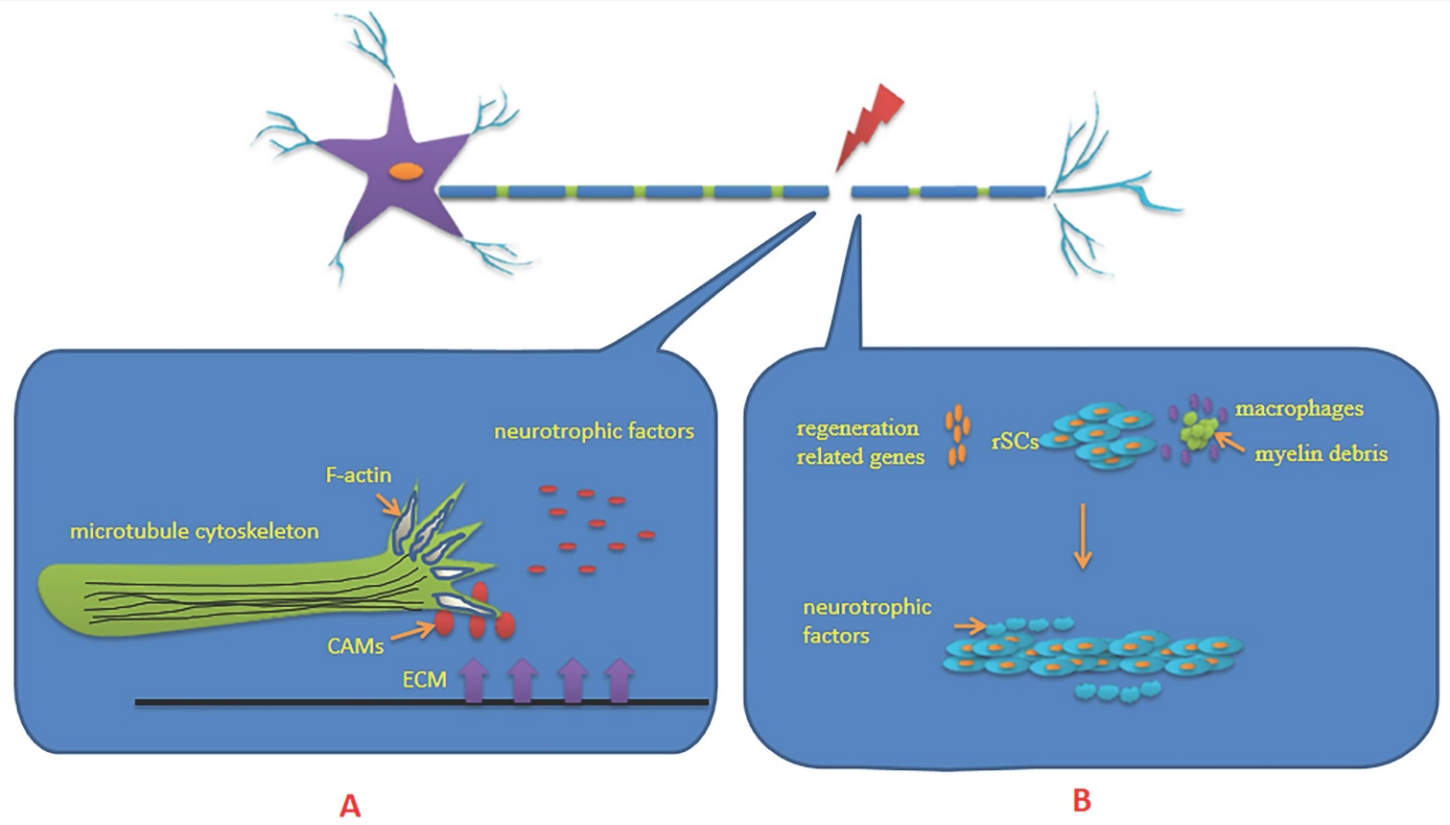
** A:** Growth cone is mostly made up of microtubule cytoskeleton and Filamentous actin (F-actin). The orientation of the advancing tip is guided by gradients of neurotrophic factors produced mainly by non-neuronal cells. The extracellular matrix (ECM) and cell adhesion molecules (CAMs) influence the state of the regenerating neurons and determine the response of the growth cone. **B:** Schwann cells (SCs) undergo dedifferentiation, proliferation, migration and remyelination after peripheral nerve injury. Differentiated Schwann cells transformed into repaired Schwann cells (rSCs). Proliferation of SCs can upregulate regeneration-associated genes, promote the aggregation of macrophages and clear myelin sheath debris. SCs form Bunger bands in the basement membrane tube and secrete a variety of neurotrophic factors. Under the support, guidance and stimulation of them, the growth cone extends from the proximal end to the distal target in the correct direction, and finally achieves nerve re-innervation.

The PNS is mainly composed of neurons and axons that connect neurons. The axons are wrapped in a myelin sheath formed by Schwann cells (SCs). After PNI, the first step to ensure successful regeneration is the formation of a growth cone on the proximal segment of severed axons, which is a highly motile mechanosensitive structure at the tip of the axon. It receives multiple extracellular guidance cues through surface receptors and transduces this information into directional movements 16. The growth cone is primarily composed of microtubule cytoskeleton and filamentous actin (F-actin). Therefore, microtubule organization and dynamics, together with axonal transport, are essential for intrinsic axon regenerative capacity 17. The orientation of the advancing tip is guided by gradients of neurotrophic factors produced mainly by non-neuronal cells, which is also known as chemotaxis 18. These neurotrophic factors include nerve growth factor, glial cell line-derived neurotrophic factor, brain derived neurotrophic factor (BDNF) and neurotrophin 3, among others. The contribution of cytoskeletal proteins to axonal regeneration is crucial. The small GTPases of the Rho family integrate the upstream signaling cues for the downstream cytoskeleton rearrangements. Receptor complexes present at the growth cone can recruit GTPases, which are needed to control the contractile capability dependent on actin-myosin interactions 19. The extracellular matrix and cell adhesion molecules influence the state of the regenerating neurons and determines the response of the growth cone 20 (**Figure 1**).

Waller degeneration is initiated at the distal segment of severed axons, which involves a series of inflammatory and immune processes including axon degeneration, myelin degeneration, SCs proliferation, macrophage aggregation and cytokine secretion. Among them, the core of the regulation is provided by SCs. SCs undergo dedifferentiation, proliferation, migration and remyelination after PNI 21 (**Figure 1**). It is widely believed that the regenerated axons which arrive at their distal targets depend largely on dedifferentiation of SCs transitioning from a transmitted state toward an immature repair phenotype. Proliferation of SCs can activate monocytes/macrophages, clear myelin sheath debris and upregulate regeneration-associated genes including growth-associated protein-43, neurotrophic factors and their receptors, neuregulin and its receptors, and others. At the same time, they form Bungner bands in the basement membrane tube and secrete a variety of neurotrophic factors. Under the support and guidance of Bungner bands, the growth cone extends from the proximal end to the distal target in the correct direction, and nerve re-innervation is finally achieved 18,22,23. The guidance and intervention of SCs dedifferentiation, proliferation, remyelination and other physiological processes on axon regeneration also play an important role as the external cause of axon regeneration. Epigenetic regulation affects axon regeneration by regulating the dedifferentiation, proliferation and remyelination of SCs.

## Classification and features of non-coding RNAs

There are many types of ncRNAs and classification methods. The two main groups are short ncRNAs and long ncRNAs, based on the length of the transcript. The former group includes small nuclear RNA, small nucleolar RNAs, miRNA, piwi-interacting RNA and small interfering RNA (siRNA). The latter group includes lncRNAs and other types. Based on the function of ncRNAs, there are housekeeping RNAs and regulatory RNAs. Ribosomal RNA, transfer RNA, small nucleolar RNA and small nucleolar RNAs are housekeeping RNAs, while miRNA, piwi-interacting RNA and siRNA are regulatory RNAs. According to subcellular location, ncRNAs can be divided into nuclear and cytoplasmic types. Many circRNAs are derived from protein-coding genes. However, circRNAs have not been proven to encode proteins, and so are also classified as non-coding RNAs. In this review, we focus on three types of ncRNAs: microRNAs (miRNAs), long non-coding RNAs (lncRNAs) and circular RNAs (circRNAs), and describe in detail their functions and roles in axonal regeneration after PNI.

The tissue-specific expression and functional roles of ncRNAs in the nervous system under physiological conditions determine their putative involvement in the pathophysiological processes of neural injury, which include immune/inflammatory responses, glial scar formation, neuronal apoptosis, cell proliferation and migration, axonal regrowth, and target organ re-innervation 24. Notably, the differential expression of ncRNAs has been found in the regeneration of PNS neurons 25, which affects the survival and regeneration of neurons and regulates phenotypic changes of the SCs.

MiRNAs are a type of endogenous regulatory ncRNAs found in eukaryotes, which are about 22 nucleotides long 26. They are initially produced in the nucleus by RNA polymerase II as long primary transcripts (pri-miRNAs), which fold into hairpins to bind to Drosha and Dicer, the RNase III families of enzymes. Finally the mature miRNAs are produced 27. They regulate gene expression by directly binding to the 3'-UTR of the target mRNA, resulting in translational repression or degradation of the mRNA. MiRNAs function in the process of neurogenesis, involving pluripotent stem cell self-renewal capability, neural progenitor cell differentiation, synaptic plasticity and rearrangement, and the release of exosomes from neural cells delivering functional nucleic acids 28. These miRNA profiles may also be influenced by the heterogeneity of injury responses in different sensory neuron subtypes. It is of vital importance to determine how different neuronal subtypes respond to injury and how the regulation of translation in different populations of sensory neurons relates to their functional recovery 5. Recent studies have also found that miRNAs after PNI significantly affected the biological behaviors of neurons and SCs, affecting neuronal survival, axonal outgrowth and phenotype modulation of SCs 29. MiRNAs play a synergistic role in well-known molecular pathways of axonal growth, inflammation, apoptosis and remyelination 30.

LncRNAs are ncRNAs with a length of 200 nucleotides or longer 31. They were originally considered to be the by-products of RNA polymerase II transcription, which did not have clear biological functions. However, studies have found multiple types of lncRNAs according to their position in the genome relative to protein-coding genes, including sense, antisense, bidirectional, intronic and intergenic lncRNAs 32. They are weakly conserved in intergenic sequences and regulate gene expression at multiple levels including epigenetic, transcriptional and post-transcriptional levels, as well as by regulating miRNAs. LncRNAs are generated in the nucleus, and some of them are exported to the cytoplasm for clipping. In fact, different localizations of lncRNAs are closely associated with the regulatory mechanism in which they participate 33,34. The regulatory mechanisms of lncRNAs are complex and diverse, including miRNA sponges, regulation of protein activity and DNA interaction with binding proteins, chromatin modification, and others 35-37. Among them, lncRNAs that function as miRNA sponges are localized to the cytoplasm, while most other lncRNAs are localized to the nucleus, where they function in chromatin epigenetic modification and regulation of DNA-protein interactions 38. So it is beneficial to make clear the subcellular localization of lncRNAs to further explore their regulatory mechanisms. In the nervous system, lncRNAs are abundant and show precisely regulated temporal and spatial expression patterns 39, which have a significant impact on many neurological diseases, including brain tumors, neurodegenerative diseases and spinal cord injury 40. In addition, they have an important regulatory function in PNI 41. LncRNAs are key regulators of neuronal differentiation, development and regeneration in the nervous system 42.

The length of circRNAs varies from 200-2000 nucleotides, with the majority having lengths of about 500 nucleotides, which are generally located in the cytoplasm. The direct ligation of the exonic downstream 5' donor site with the upstream 3' acceptor site results in a closed loop structure, which makes circRNAs resistant to degradation by exonuclease RNaseR and helps them maintain high stability and conserved sequence 43. According to their origin, circRNAs may be composed of exons, introns and exon-intron structures (ElciRNA) 44. The circRNAs transcribed from exons contain miRNA binding site elements and have an miRNA sponging effect similar to that of lncRNAs. They regulate gene expression through competitive binding with corresponding miRNA site 45,46. The circRNAs transcribed from introns are mostly combined with RNA polymerase III to promote gene transcription. The circRNAs transcribed from introns and exons in cell nuclei can interact with small ribosomal U1 snRNP to form a complex with RNA polymerase II in the promoter region to promote gene expression 47. Moreover, circRNAs are efficiently translated in living human cells to produce abundant protein products by a rolling circle amplification mechanism 48. Through the above biological functions, circRNAs are thus able to regulate neuron regeneration and repair.

## Non-coding RNAs in peripheral nerve regeneration

### MiRNAs in peripheral nerve regeneration

#### Neuron survival and regeneration

The survival of injured neurons is a necessary prerequisite for activating their inherent regeneration ability. It has been determined that cell body damage or even cell death will occur around the lesion site after PNI 49. In addition to intrinsic neurotrophic factors, such as BDNF, ciliary neurotrophic factor and fibroblast growth factor-2, miRNAs also play important roles in the survival of injured neurons. Downregulated miR-192-5p can promote the expression of X-linked inhibitor of apoptosis protein (XIAP) to reduce apoptosis of nerve cells and promote the regeneration of sciatic nerve injury 50. Moreover, overexpression of miR-210 inhibited cell apoptosis by inhibiting its target ephrin-A3, thereby promoting sensory axon regeneration 51. Inhibition of endogenous miR-26a in sensory neurons impaired axon regeneration by increased expression of glycogen synthase kinase 3β. It was also found that a new miR-26a-glycogen synthase kinase 3β-Smad1 pathway could regulate axon regeneration 52. Smad1, a key member of the pathway, is a well-known axon regeneration-related transcription factor 53. During biological processes, epigenetic modification is frequently a pivotal approach to modulate gene expression. Researchers found that miR-9 targets FoxP1 directly and regulates mammalian axon regeneration post-injury. High level of endogenous miR-9 in sensory neurons inhibited axon regeneration 54.

#### Schwann cells

As mentioned above, SCs promote nerve repair through dedifferentiation, proliferation, migration, and remyelination after PNI. Therefore, it is necessary to discuss how miRNA regulates SCs. Damage or death of SCs induced by injuries adversely affects nerve regeneration. Research identified that miR-1b was downregulated following sciatic nerve injury and NDRG3 was verified as a direct target gene of miR-1b. Overexpression of miR-1b decreased the proliferation and migration as well as increased the apoptosis of SCs, while high expression of NDRG3 reversed the effect of miR-1b overexpression 55. In addition, other studies have found that overexpressed miR-1b inhibited the proliferation, migration, and promoted apoptosis of SCs by inhibiting BDNF 56.

Functional recovery after PNI relies on the reprogramming of differentiated SCs into repaired SCs. Transition into a repair phenotype requires expression of c-Jun and Sox2, which transcriptionally mediates inhibition of the differentiation program of myelination and activates a non-cell-autonomous repair program 4. Overexpression of miRNA-21 leads to downregulation of PTEN and activation of phosphatidylinositol-3 kinase (PI3K). Increased expression of miRNA-21 promotes axon regeneration and functional recovery after nerve injury 57.

Accurate path-finding is essential for axon growth. Ntn1 and its receptor Dcc have profound functions in axon guidance. Researchers have found that miR-9 decreases the levels of Dcc and inhibits SCs migration. miR let-7 and miR-9 decrease the protein levels of Ntn1 and Dcc, respectively, thereby inhibiting axonal regeneration. In addition, a potential regulatory network has been identified, involving let-7, miR-9, Ntn1, Dcc and other related molecules, such as RNA binding protein Lin-28 homologous protein A, SRC proto-oncogene nonreceptor tyrosine kinase and the transcription factor NF-κB 58. A recent study found that ulinastatin could significantly inhibit the expression of let-7 and increase the expression of nerve growth factor, thereby enhancing the proliferation and migration of SCs and reducing oxidative stress. Ulinastatin is a serine protease inhibitor with various anti-inflammatory and antioxidant biological activities. It can upregulate nerve growth-associated protein-43 and NF200 and myelin formation-related proteins MAG and PMP22 59. These findings suggest a possible approach for the treatment of PNI. In addition, miR-34a and miR-148b overexpression evidently inhibited the proliferation and migration of SCs by targeting contactin-2 and calreticulin, respectively 60,61. At the same time, a large number of miRNAs have been shown to promote the proliferation and migration of SCs. The upregulated miR-sc14 acts on fibroblast growth factor receptor 2 and miR-3099 acts on multiple targets including Aqp4, St8sia2, Tnfsf15 and Zbtb16 to promote the proliferation and migration of SCs 62,63. Mir-29a-3p inhibits proliferation and migration of SCs by negatively regulating peripheral myelin protein 22 (PMP22), which is closely correlated with myelin sheath formation 64.

Remyelination after PNI is a key step in axon regeneration. A study found that miR-485-5p overexpression targets Cdc42 and rac1 to inhibit SCs proliferation and myelination 65. Upregulation of miR-221-3p inhibited the myelination of SCs by target inhibiting Nab1 66. The enhanced expression of miR-30c significantly increased the amount of myelin-associated protein in co-cultured dorsal root ganglion (DRG) and SCs, suggesting that miR-30c functions to promote SCs myelination after PNI 67 (**Table 1**).

### LncRNAs in peripheral nerve regeneration

#### Neuron survival and regeneration

In recent years, studies have found that a large number of lncRNAs are differentially expressed after PNI and play an important role in peripheral nerve regeneration. It was found that silencing BC089918 and uc.217 by siRNA significantly promoted DRG neurite outgrowth 68,69. Sox11 is involved in the regulation of multiple genes including adhesion molecules, cytoskeleton elements, growth factors, cytokines, neuropeptides and other molecules associated with regeneration. Silc1 is an lncRNA with a conserved sequence and is highly expressed in neuronal tissues. Experiments indicated that Silc1 regulated nerve regeneration by cis-activating Sox11 70.

LncRNA-mRNA-miRNA co-expression network analysis has become an important research tool. Researchers found that the expression of lncRNA-GM4208 and lncRNA-GM30085 was upregulated in the injured mouse sciatic nerve, while the expression of miRNA-138, miRNA-500, miRNA-6538 and miRNA7239 was downregulated. It was hypothesized that the lncRNA, miRNA and mRNA formed a competing endogenous RNA (ceRNA) network to regulate neuron regeneration after sciatic nerve injury 71. LncRNA-mRNA-miRNA co-expression network analysis suggested lncRNA Arrl1 inhibited axon regeneration. A potential mechanism was proposed in which Arrl1 is a ceRNA acting as an miRNA molecular sponge which absorbs miR-761, an inhibitor of cyclin-dependent kinase inhibitor 2B (Cdkn2b), and thus, upregulates Cdkn2b (the inhibition of Cdkn2b significantly facilitates DRG axon regeneration) 72.

#### Schwann cells

The proliferation and migration of SCs have a profound impact on axon regeneration after PNI. Studies have shown that lncRNAs can act on SCs after PNI and play an important role in peripheral nerve regeneration. LncRNA snoRNA hostgene16 (SNHG16) is located on human chromosome 17 17q25.1 (13,14). Studies have reported that up-regulated SNHG16 promotes the expression of STAT3, which is a target of miR-93-5p, through endogenous competitive sponge adsorption of miR-93-5p, thus increasing SCs proliferation and migration 73. Similarly, up-regulated lncRNA MALAT1 elevates BDNF by sponging miR-129-5p 74. LncRNA Loc680254 was significantly upregulated after PNI, inducing SCs proliferation, thereby promoting axon regeneration and functional recovery. It may play a regulatory role by acting as an miRNA sponge to affect the expression of Psrc1 and Ska1. Psrc1 and Ska1 cooperate with each other to affect the cell cycle and proliferation 75. NEAT1 is a lncRNA that is upregulated in many tumor tissues. It promotes tumor cell proliferation and migration. MiR-34a is a tumor suppressor targeting NEAT1 in SCs. Satb1, an oncogene, is also the target gene of miR-34a, and there is a negative regulatory effect between Satb1 and miR-34a. Studies have shown that NEAT1 is upregulated after PNI. As a ceRNA of miR-34a, NEAT1 negatively regulates the expression of miR-34a, further increasing the expression of Satb1, and promoting the proliferation and migration of SCs, as well as peripheral nerve regeneration 76. Microarrays were used to screen the upregulated lncRNAs after sciatic nerve injury. It was found that lncRNA BC088259 showed the most significant upregulation. Further study found that BC088259 could interact with vimentin and regulate the migration of SCs after sciatic nerve injury. Vimentin is a major intermediate filament protein involved in cell development, attachment and migration. Silencing the expression of vimentin significantly inhibited the migration of SCs 77. Knockdown of lncRNA Bc088327 was found to inhibit the cell viability and induce cell apoptosis and S-phase arrest in the SCs. LncRNA Bc088327 may also synergize with Hereglin-1β, which participates in nerve regeneration 78. In addition to the above lncRNAs, the downregulation of some lncRNAs can also enhance the proliferation and migration of SCs. For instance, the downregulation of lncRNA MEG3 increased the proliferation and migration of SCs and facilitated nerve regeneration and functional recovery through the PTEN/PI3K/AKT pathway 79. Silencing lncRNA TNXA-PS1 could induce SCs migration to promote axon regeneration. A likely mechanism is that TNXA-PS1 may attenuate the inhibitory effect of miR-24-3p/ miR-152-3p on dual-specificity phosphatase 1 (DUSP1) by sponging miR-24-3p/miR-152-3p. After nerve injury, the downregulation of TNXA-PS1 decreased expression of DUSP1 and promoted the migration of SCs 80. It has been confirmed that overexpression of lncRNA MSTRG.24008.1 activates nucleotide-binding oligomerization domain-like pyrin domain containing 3 (NLRP3) and myelin and lymphocyte protein (MAL) via miR-331-3p to inhibit the proliferation of SCs at the injured site and further reduce the regeneration of the axon 81 (**TABLE 2**).

### CircRNAs in peripheral nerve regeneration

In recent years, circRNAs have been increasingly studied in nerve injury, which have a sponge-like effect on miRNAs. A bioinformatics database of circRNAs has not been completely established, so research progress on circRNAs has been relatively slow. It was found that circRNA 012142 was significantly upregulated after PNI. CircRNAs act as an miRNA sponge to inhibit miRNA activity through an interacting network with miRNAs. The circRNA/miRNA/mRNA network may be involved in multiple biological functions during axonal regeneration and degeneration 82. CircRNA-Spidr, which is derived from the Spidr gene, has its highly expression in the ovary and functions in DNA double-strand break repair through homologous recombination, thus helping to maintain the integrity of the genome. In addition, CircRNA-Spidr is a circRNA enriched in the cytoplasm of DRG neurons. Experiments have shown the downregulation of CircRNA-Spidr can regulate the PI3K-Akt signaling pathway to inhibit axon regeneration of DRG neurons after sciatic nerve injury 83. Researchers also found that downregulated circRNA.2837 alleviated sciatic nerve damage by inducing autophagy in a rat model of sciatic nerve injury. The mechanism may be that ceRNA regulates the autophagy of neurons by sponging members of the miR-34 family to protect neurons from injury 84.

CircRNA-Ankib1 is also associated with the proliferation of SCs. Overexpression of CircRNA-Ankib1 impairs SCs proliferation and axon regeneration after sciatic nerve injury. CircRNA-Ankib1 can directly bind with miR-423-5p, miR-485-5p, and miR-666-3p and function as their sponge to regulate Cyp26b1 expression 85 (**Table 2**).

## Discussion

**Figure 2:**
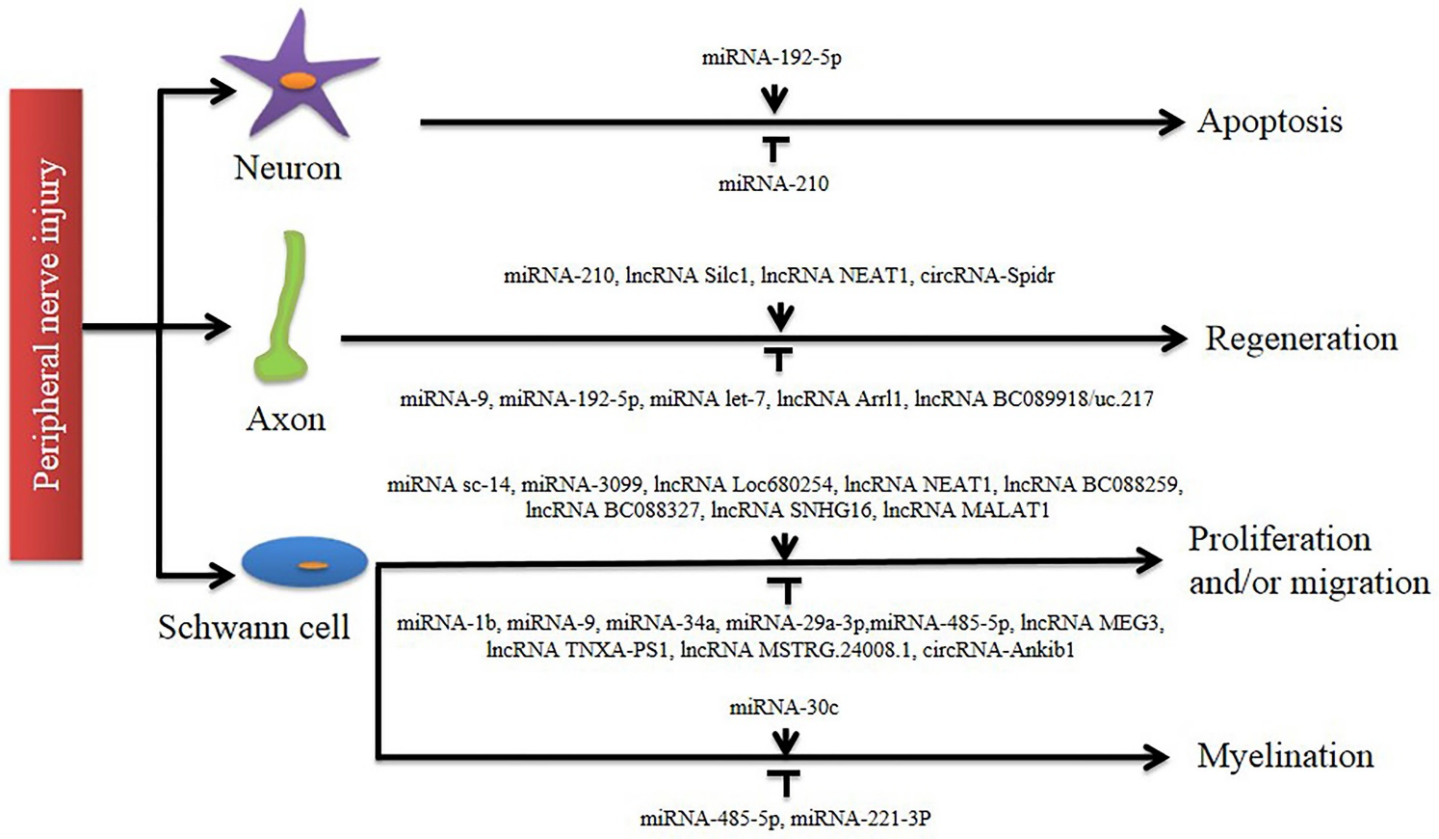
Involvement of non-coding RNAs in peripheral nerve injury. A number of studies identify that different non-coding RNAs exert regulatory functions in axon regeneration and Schwann cell phenotype modulation (including proliferation, migration, and myelination). Their distinct roles in post-nerve-injury changes are indicated by an arrow line (for activation) and a T-shaped line (for inhibition), respectively.

In this review, we first described the cellular and molecular mechanisms of axonal regeneration after PNI. A series of studies have shown that after PNI, a large number of ncRNAs become involved, and their differential expression suggests that they play an important role in the repair of the nervous system (**Figure 2**). Then, we explained the respective functions of miRNAs, lncRNAs and circRNAs in detail. In addition to the above, there are many ncRNAs that also play an important role, such as snRNA which is involved in splicing mRNA, snoRNA which guides post-transcriptional RNA modifications of other ncRNAs, piRNA which is important in maintaining genomic stability by targeting and inhibiting repetitive transposable elements, and siRNA which plays a role in inducing post-transcriptional gene silencing 86.

Increasing evidence has shown that ncRNAs are involved in a variety of cellular processes and are associated with human diseases. This indicates that miRNAs, lncRNAs and circRNAs may be potential clinical diagnostic biomarkers and therapeutic targets. More importantly, these ncRNAs are easily detectable in body fluids. However, nerve injury repair is a very complex process involving multiple systems and multiple signaling pathways. Therefore, it's of vital importance to explore the source, characteristics, functions and regulatory mechanisms of ncRNAs in order to promote their clinical application. In previous studies, researchers have found that differentially expressed ncRNAs were derived from DRG neurons or SCs after sciatic nerve injury 68,69,87,88. In addition, some ncRNAs can also be carried by exosomes. Exosomes originating from SCs, macrophages and mesenchymal stem cells can promote peripheral nerve regeneration 89. However, more of them are endogenous, which is a significant factor in the regeneration process in PNS 52, 54.

Currently due to the structural complexity of the nervous system in humans which cannot be reproduced in vitro, researchers have employed the murine sciatic nerve injury model, which is the most used experimental paradigm for the pre-clinical investigation of peripheral nerve regeneration 90. The reason is that the size of sciatic nerve is large, which facilitates surgery, and data from previous comparable studies is readily available. However, due to limited size of the defects that can be created and the speed and quality of nerve regeneration, PNIs seen in the clinic are difficult to reproduce in murine sciatic nerve injury models. At present, there is no single experimental model that is superior to others. Hence, researchers need to carefully evaluate their specific requirements and expertise, choosing the most suited pre-clinical model 91.

**Figure 3:**
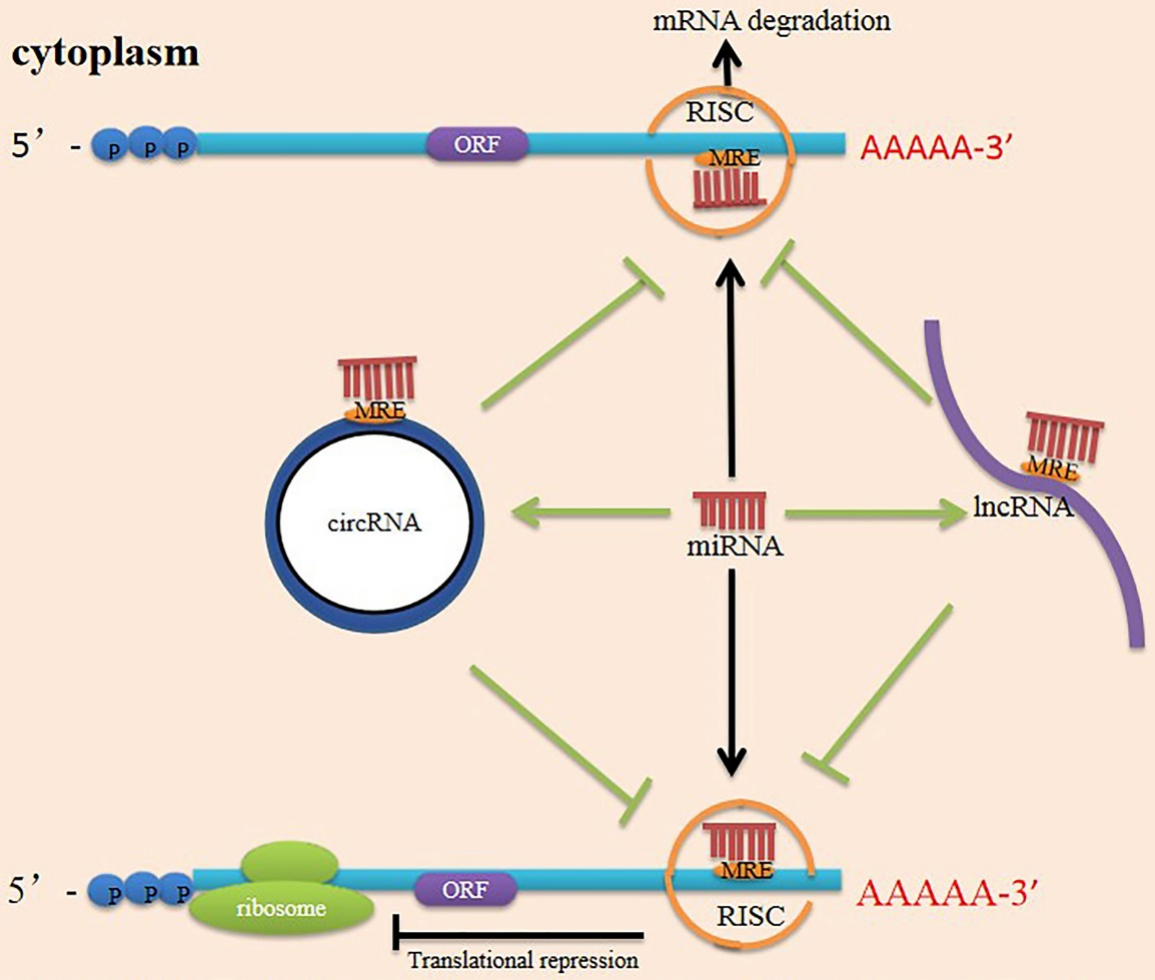
Mechanism scheme of ceRNA network. There are multiple miRNA response elements (MRE) on the transcription of coding genes (mRNA), and miRNA can bind to mRNA through MRE, forming a RISC (RNA-induced silencing complex) in their 3' untranslated regions of the target mRNA. As a result, the miRNA silences the gene through mRNA degradation or transcriptional repression. Both lncRNAs and circRNAs have many MRE and serve as miRNA sponges in cells. They compete with mRNA for opportunities to bind with miRNAs. In this way, lncRNAs and circRNAs remove the miRNA's inhibition to the target mRNA, and the expression of the mRNA increases as a consequence. This mechanism is called competing endogenous RNA (ceRNA). ORF (open reading frame).

Currently, ceRNAs have attracted considerable attention, suggesting a new direction for future research. Researchers initially proposed the ceRNA hypothesis, positing that RNAs can engage in crosstalk with each other and manipulate biological functions independently of protein translation 92. MiRNAs recognize miRNA response elements (MREs) on the 3' UTR of mRNA and silence target genes by repressing mRNA translation or degrading the mRNA 93. LncRNAs and circRNAs contain MREs and can use MREs to bind miRNAs. They function as ceRNAs to sponge miRNAs and reduce miRNAs activity, impairing the interaction between miRNAs and their targeted genes 94 (**Figure 3**). The earliest ceRNA mechanisms and network construction have been reported in cancers. Numerous studies have reported that the ceRNA mechanism operates in various diseases and that ceRNA expression patterns vary under different tissue and cellular conditions 95. One study has connected differentially expressed lncRNAs with differentially expressed mRNAs after PNI to construct an endogenous competitive network of LIF and Hmox1 and describe the function of the mRNA-miRNA-lncRNA-ceRNA network following PNI 96. CircRNAs can also function as miRNA or lncRNA sponges to indirectly regulate mRNA expression through a complex RNA communication network in nerve regeneration 25. However, the interactions between these networks will require further documentation in PNI.

In recent years, more than 170 different modifications have been identified in RNA 97. N^6^-methyladenosine (m^6^A) is the most abundant form of methylation modification in mRNA, which is methylated at the N^6^ position of the adenosine base. It is dynamically regulated by methyltransferase and demethylase and binding proteins. M^6^A modification regulates cell proliferation, apoptosis and the cell cycle by promoting the maturation of miRNA, the translation and degradation of circRNA, and the stability of lncRNA 98. When the mammalian nervous system is damaged, m^6^A is involved in regulating protein synthesis and axon regeneration. M^6^A can control the translation process by regulating mRNA, through which axon regeneration and functional recovery is affected 99. To the best of our knowledge, the role of m^6^A methylation in regulating ncRNAs has not been examined in PNI, and therefore provides a possible direction for future research.

Exosomes are small secretory vesicles with a diameter of 30-150 nm that act as intercellular communication tools to transfer cytokines, mRNA, miRNA, DNA and proteins between cells, thereby causing biological reactions in recipient cells 100. During peripheral nerve repair, exosomes serve as important modulators to provide a suitable microenvironment for regeneration as well as regulate communication between different cell types. Exosomes have been shown in a variety of studies to bind to recipient cells through protein molecules on their surfaces, enter recipient cells and release contents such as miRNAs, or trigger signal transduction to affect the physiological functions of recipient cells 101. Emerging studies have shown that ncRNA-containing exosomes, which stem from sensory neurons, mesenchymal stem cells, SCs or macrophages, can mediate intercellular communication by transferring these types of cargo and stimulating axonal regeneration 102. It has been shown that after PNI, miR-21-5p is upregulated in DRG neurons. Sensory neuron-derived exosomes are readily phagocytosed by macrophages, and an increase in miR-21-5p expression promotes a pro-inflammatory phenotype, which contributes to sensory neuron-macrophage communication after damage to the peripheral nerve 103. Exosomal modification is an important part of the SCs repair program. Overexpression of miR-21 leads to downregulation of PTEN and activation of PI3K, which is responsible for the pre-regeneration ability of SC exosomes and promotes axon regeneration and functional recovery after nerve injury 57. The extracellular vesicles produced by mechanically stimulated SCs contain miR-23b-3p, whose overexpression inhibits Nrp1 expression and plays an active role in axon regeneration 104. This provides a new idea for the further study of axon regeneration in PNI. We need more exploration of ncRNAs to expand our thinking. This includes further exploration of the mechanism by which m^6^A methylation affects axon regeneration through the regulation of ncRNAs and exosomes carrying ncRNAs to complete intercellular transmission and participate in axon regeneration.

Although considerable progress has been made in understanding the underlying mechanisms of peripheral nerve regeneration and how these mechanisms can be harnessed to promote regeneration of the PNI, there are still many challenges to overcome in developing treatments that achieve complete regeneration and functional recovery of axons. At present, the clinical treatment of PNI mainly includes nerve repair, autologous nerve transplantation and artificial nerve conduits, among other approaches. These methods can improve the repair effect and reduce the formation of neuroma and neuropathic pain. However, functional recovery after PNI is often unsatisfactory 105,106. We will need to use combination therapy to improve the cure rate of patients. NcRNAs in the nervous system have tissue-specific expression and regulate the expression of regeneration-associated genes after PNI. Therefore, they have great potential for gene therapy in axonal regeneration after nerve injury. NcRNA-based therapeutics have rapidly emerged in recent years as a promising alternative strategy in neural regenerative medicine 28. The clinical value of ncRNAs in nerve regeneration therapy and a method to select the appropriate vector to ensure the successful delivery of ncRNAs to the desired target also need to be further explored. While we have made a good start, we still need to translate current animal studies into clinical applications as soon as possible.

In conclusion, ncRNAs provide us with further clues to understand the potential mechanism of peripheral nerve regeneration. More efforts are needed to promote the application of ncRNAs as a therapeutic target in PNI.

## Figures and Tables

**Table 1:** Summary of the functions and mechanisms of miRNAs in peripheral nerve injury.

	Human orthologs	Injury model	Expression	Target	Function	Reference
miRNA-192-5p		Rat sciatic nerve crush	Upregulation	XIAP	Inhibition of miRNA-192-5p promote neurite outgrowth and decrease neurons apoptosis	50
miRNA-210		Mouse sciatic nerve crush		EFNA3	miRNA-210 overexpression promote axon regeneration and inhibit apoptosis	51
miRNA-26a	Human	Mouse sciatic nerve crush		GSK3β	Inhibition of miRNA-26a reduce axon regeneration	52
miRNA-9	Human	Mouse sciatic nerve crush		FoxP1	miRNA-9 overexpression inhibit axon regeneration	54
miRNA-1b	Human	Rat sciatic nerve transection	Downregulation	NDRG3	miRNA-1b overexpression inhibit proliferation, migration, and promote apoptosis of SCs	55
miRNA-1b	Human	Rat sciatic nerve transection	Upregulation	BDNF	miRNA-1b overexpression inhibit proliferation, migration, and promote apoptosis of SCs	56
miRNA let-7		Rat sciatic nerve crush		Ntn1	miRNA let-7 inhibit axon regeneration	58
miRNA-9	Human	Rat sciatic nerve crush		Dcc	miRNA-9 inhibit SCs migration and axon regeneration	58
miRNA-34a	Human	Rat sciatic nerve crush	Downregulation	CNTN2	miRNA-34a inhibit SCs proliferation and migration	60
miRNA sc-14		Rat sciatic nerve transection	Differential expression	FGFR2	miRNA sc-14 overexpression promote SCs proliferation and migration	62
miRNA-3099		Rat sciatic nerve crush	Upregulation	Aqp4/ St8sia2/ Tnfsf15 /Zbtb16	miRNA-3099 overexpression promote SCs proliferation and migration	63
miRNA-29a-3p	Human	Rat sciatic nerve crush	Upregulation	PMP22	miRNA-29a-3p overexpression inhibit SCs proliferation and migration	64
miRNA-485-5p	Human	Mouse sciatic nerve crush	Downregulation	Cdc42 and Rac1	miRNA-485-5p overexpression inhibit SCs proliferation and myelination	65
miRNA-221-3p	Human	Rat sciatic nerve resect		Nab1	miRNA-221-3p overexpression inhibit SCs myelination	66
miRNA-30c		Rat sciatic nerve transection	Differential expression		miRNA-30c promote SCs myelination	67

**Table 2:** Summary of the functions and mechanisms of lncRNAs and circRNAs in peripheral nerve injury.

	Human orthologs	Injury model	Expression	Target	Function	Reference
lncRNA BC089918/uc.217		Rat sciatic nerve transection	Downregulation		Inhibition of lncRNA BC089918/uc.217 promote axon regeneration	68,69
lncRNA Silc1	Human	Mouse sciatic nerve crush	Upregulation	Sox11	lncRNA Silc1 promote axon regeneration	70
lncRNA Arrl1		Rat sciatic nerve crush	Downregulation	miRNA-761a/Cdkn2b	lncRNA Arrl1 inhibit neurite outgrowth	72
lncRNA SNHG16	Human	Rat sciatic nerve transection	Downregulation	miRNA-93-5p/STAT3	lncRNA SNHG16 overexpression promote SCs proliferation and migration	73
lncRNA MALAT1	Human	Mouse sciatic nerve crush	Upregulation	miRNA-129-5p/BDNF	lncRNA MALAT1 overexpression promote SCs proliferation and migration	74
lncRNA Loc680254		Rat sciatic nerve crush	Upregulation	Psrc1/Ska1	lncRNA Loc680254 overexpression promote SCs proliferation	75
lncRNA NEAT1	Human	Mouse sciatic nerve crush	Upregulation	miR-34a/Satb1	lncRNA NEAT1 overexpression promote SCs proliferation, migration and axon regeneration	76
lncRNA BC088259		Rat sciatic nerve crush	Upregulation	Vimentin	lncRNA BC088259 overexpression promote SCs migration	77
lncRNA BC088327		Rat sciatic nerve transection	Upregulation		Inhibition of lncRNA BC088327 suppress SCs proliferation and promote apoptosis	78
lncRNA MEG3	Human	Rat sciatic nerve transection	Upregulation	PTEN/PI3K/AKT	Inhibition of lncRNA MEG3 promote SCs proliferation and migration	79
lncRNA TNXA-PS1		Rat sciatic nerve crush	Downregulation	miRNA-24-3p/miRNA-152-3p/Dusp1	Inhibition of lncRNA TNXA-PS1 promote SCs migration	80
lncRNA MSTRG.24008.1		Rat sciatic nerve crush	Upregulation	miRNA-331-3p/NLRP3/MAL	Inhibition of lncRNA MSTRG.24008.1 promote SCs proliferation	81
circRNA-Spidr	Human	Rat sciatic nerve crush	Upregulation	PI3K-Akt	circRNA-Spidr promote axon regeneration	83
circRNA.2837		Rat sciatic nerve crush	Downregulation	miR-34	Inhibition of circRNA.2837 induce neurons autophagy	84
circRNA-Ankib1		Rat sciatic nerve crush	Downregulation	miR-423-5p/miR-485-5p/miR-666-3p/ Cyp26b1	circRNA-Ankib1 inhibit SCs proliferation and axon regeneration	85
